# The toxic effect of cytostatics on primary cilia frequency and multiciliation

**DOI:** 10.1111/jcmm.14487

**Published:** 2019-06-17

**Authors:** Alžběta Filipová, Daniel Diaz Garcia, Aleš Bezrouk, Dana Čížková, Josef Dvořák, Stanislav Filip, Justin Sturge, Zuzana Šinkorová

**Affiliations:** ^1^ Department of Radiobiology, Faculty of Military Health Sciences in Hradec Králové University of Defence Hradec Králové Czech Republic; ^2^ Department of Clinical Biochemistry and Diagnostics University Hospital Hradec Králové Czech Republic; ^3^ Department of Medical Biophysics, Faculty of Medicine Charles University Hradec Králové Czech Republic; ^4^ Department of Histology and Embryology, Faculty of Medicine Charles University Hradec Králové Czech Republic; ^5^ Department of Oncology, Thomayer Hospital Charles University Prague Czech Republic; ^6^ Department of Oncology and Radiotherapy, Faculty of Medicine Charles University Hradec Králové Czech Republic; ^7^ Department of Biomedical Sciences, Faculty of Health Sciences University of Hull Hull UK

**Keywords:** doxorubicin, multiple cilia, primary cilium, triple-negative breast cancer

## Abstract

The primary cilium is considered as a key component of morphological cellular stability. However, cancer cells are notorious for lacking primary cilia in most cases, depending upon the tumour type. Previous reports have shown the effect of starvation and cytostatics on ciliogenesis in normal and cancer cells although with limited success, especially when concerning the latter. In this study, we evaluated the presence and frequency of primary cilia in breast fibroblasts and in triple‐negative breast cancer cells after treatment with cytostatics finding that, in the case of breast fibroblasts, primary cilia were detected at their highest incidence 72 hours after treatment with 120 nM doxorubicin. Further, multiciliated cells were also detected after treatment with 80 nM doxorubicin. On the other hand, treatment with taxol increased the number of ciliated cells only at low concentrations (1.25 and 3.25 nM) and did not induce multiciliation. Interestingly, triple‐negative breast cancer cells did not present primary cilia after treatment with either doxorubicin or taxol. This is the first study reporting the presence of multiple primary cilia in breast fibroblasts induced by doxorubicin. However, the null effect of these cytostatics on primary cilia incidence in the evaluated triple negative breast carcinomas cell lines requires further research.

## 1 INTRODUCTION

Primary cilium, an organelle found on nearly every cell in the human body, typically serves as a mechanical sensory tool; further, it is also involved in cell proliferation and embryonic development. This organelle is dynamically regulated during the cell cycle, appearing during the G0/G1 phases and resorbed prior to mitosis.^1^ The exact solubilization moment of primary cilia is determined by cell type and the expression of genes affecting resorption, such as Aurora A, Plk1 and TcTex‐1.^2^, ^3^ Usually, a cell has only one primary cilium which, as previously mentioned, is involved in morphogenesis, cell proliferation and differentiation signalling.^4^, ^5^ Should there be a multiplication of centrosomes, a higher number of primary cilia will appear on the surface of cells as well, often bearing the same length and construction design, and spawning from the same ciliary pocket; however, the presence of multiple cilia has been mostly recorded in solid tumours^6^, ^7^ after exposure to ionizing irradiation^8^, ^9^ or in ciliopathies.^10^ Typically, primary cilia are always observed in myoepithelial cells and fibroblasts and with low incidence in luminal epithelium cells. Regarding cancer cells, a study of 26 breast cancer biopsy samples revealed the presence of primary cilia only on exceptional cases, especially in epithelial cells.^11^

Basal subtype B tumours, a classification of triple negative breast carcinomas (TNBCs), are characterized by the absence of oestrogen, progesterone and Her2/neu receptors,^12^ as well as by the rare presence of primary cilia. Among breast cancer tumours, TNBC has an estimated incidence of 10%‐20%, although from a histological point of view TNBCs are little differentiated and are often included in basal‐like subgroups. From a clinical standpoint, these tumours are frequently resistant to treatment, have quick progression, low 5‐year survival rate, increased local recurrence and are highly metastatic. This kind of tumours can be observed at any age; however, they mostly occur accompanied by BRCA1 mutations in younger women (>40 years of age).^13^ Chemotherapy is the treatment of choice for triple‐negative breast cancer patients, of which Doxorubicin and Taxol are the standard chemotherapeutic agents used as anticancer therapy in combination with α‐HER2/neu receptor targeted therapy. Doxorubicin belongs to the anthracyclines group, whereas Taxol is considered as a taxane. The former is an effective intercalating cytotoxic agent used in the treatment of various tumour types and commonly used in combination with the latter in adjuvant and neoadjuvant therapeutic strategies for breast cancer patients.^14^

Previous research on the effect of cytostatics on primary cilia has focused in the effects of Taxol over the elongation and shortening of primary cilia. In a study by Sharma et al., Taxol was shown to block the emergence of primary cilia in mammalian cell cultures.^15^ However, low concentrations result in an increased quantity of free tubulin subunits in the cytosol, leading to enlarged primary cilia.^16^, ^17^ Ongoing research highlights two important questions in the cell biology of cancer and primary cilia: (a) the significance of having primary cilia in normal cells and (b) the loss of primary cilia in cancer cells and its relation to drug resistance.^18^ Therefore, the increased primary cilia frequency induced by cytostatics could be used in other studies trying to assess the toxicity of these drugs.

## 2 MATERIALS AND METHODS

### 2.1 Cell culture

Unless otherwise stated, all standard chemicals and antibodies were purchased from Sigma‐Aldrich, Czech Republic. In this study, TNBC cell lines BT‐549 (ATCC, USA) and MDA‐MB‐231 were used (kindly supplied by Mgr. Jaroslav Truksa, Ph.D., Laboratory of Tumor Resistance, Institute of Biotechnology CAS, Prague), as well as skin fibroblasts. BT‐549 cells were cultured in DMEM 10% FBS (PAA, USA), 2% glutamine (Gibco, UK), 1% penicillin/streptomycin (Gibco, UK), 0.023 IU/mL insulin and incubated in a 5% CO_2_ atmosphere at 37°C. MDA‐MB‐231 and fibroblasts were cultured in DMEM, 10% FBS (PAA, USA), 2% glutamine (Gibco, UK), 1% penicillin/streptomycin (Gibco, UK) and incubated in a 5% CO_2_ atmosphere at 37°C. All cell lines were used until 10th to 12th passage and medium was replaced every 2 days for all experiments.

### 2.2 Skin fibroblasts

Human fibroblasts were isolated from skin biopsies obtained in accordance and approved by the Ethics Committee of the University Hospital Hradec Kralove, Czech Republic and the European Ethics committee under the directive approved on 10 July 2014 (Reference/license number: 201407 S12P). Donor patients signed an informed consent allowing us to work with the obtained samples. To isolate the fibroblasts, skin biopsies were kept in a basic solution (30 mL/2 g of tissue) after surgery and transported to the laboratory, where the biopsies were washed in PBS and the subcutaneous tissue was removed. Each individual tissue sample was incubated in 5 mL of a 2 U/mL dispase solution (Gibco, UK) for 18 hours at 4°C. After incubation, the samples were washed three times in PBS at room temperature (22°C). The dermis was separated from the epidermis using tweezers, washed in PBS and cut into small sections (<5 mm^2^). Then, the tissue was incubated in 10 mL of digestion solution (1 g tissue/10 mL) in a rotating incubator at 37°C/6 g. After 4 hours, the samples were filtered through a 40 μm strainer into a new sterile 50 mL tube. The cell number was determined from this suspension and centrifuged for 10 minutes/150 g. The supernatant was decanted and the cell pellet was resuspended in culture media, plated in a T‐75 flask (1 × 10^5^ cells per flask) and incubated in a 5% CO_2_ atmosphere at 37°C for 14 days.

### 2.3 Cytostatic drugs

Doxorubicin and Taxol were dissolved in 0.5% DMSO and kept in 1 mM stock solutions. Doxorubicin and Taxol were diluted in culture media before use at a ratio of 1:100 and 1:1000 respectively.

### 2.4 Cell treatment and immunofluorescence

MDA‐MB‐231, BT‐549 and fibroblasts cells were cultured in 6‐well plates at a density of 3 × 10^5^ cells per well and incubated at 37°C/5% CO_2_ for 24 hours, each well contained a gelatine‐coated coverslip. After this period, the cells were treated with Doxorubicin (10, 20, 40, 80, and 120 nM) or Taxol (1.25, 3.25, 5.25, 6.25 and 12.5 nM) for 72 hours. Control cells were kept under the same conditions in culture media with or without DMSO (0.5%). After treatment, the cells were fixed with 4% paraformaldehyde for 10 minutes at room temperature and washed three times with PBS. Immunostaining was performed as follows: cells were blocked with goat serum (Jackson Immunoresearch, USA) 1:20 for 30 minutes; anti‐acetylated tubulin 1:800 for 1 hour; anti‐gamma tubulin 1:500 for 1 hour; Cy3‐conjugated donkey anti‐mouse secondary antibody 1:300 for 45 minutes in the dark; Alexa 488 conjugated donkey anti‐rabbit secondary antibody (JacksonImmunoresearch, USA) 1:300 for 45 minutes in the dark. Imaging analysis was performed with a Nikon Eclipse fluorescence microscope (Prague, Czech Republic) observed with an oil‐immersion 60× objective. All experiments were performed in triplicate.

### 2.5 Cytotoxicity test

A WST‐1 test (Roche; Basel, Switzerland) was used to determine cell viability after treatment with Doxorubicin (10, 20, 40, 80, and 120 nM) or Taxol (1.25, 3.25, 5.25, 6.25 and 12.5 nM). All cell lines were plated and treated in a 96‐well‐plate (1 × 10^3^ cells per well), and incubated at 37°C/5% CO_2_ for 24 hours. After 72 hours, 10 µL of WST‐1 reagent was added and incubated for 3 hours at 37°C/5% CO_2_ and analysed in a Tecan SpectraFluor Plus spectrometer (Tecan Austria GmbH; Grödig, Austria) at a wavelength of 440 nm. All experiments were performed in triplicate.

### 2.6 Transmission electron microscopy (TEM)

Fibroblasts were fixed in 3% glutaraldehyde (in 0.1 M cacodylate buffer, pH 7.2) for 5 minutes at 37°C and then for 3 hours at room temperature, washed in cacodylate buffer (0.1 M, pH 7.2) and post‐fixed in 1% OsO4 (in 0.1 M cacodylate buffer, pH 7.2) for 1 hour at room temperature. After rinsing in cacodylate buffer (0.1 M, pH 7.2), the cells were dehydrated in graded alcohols (50%, 75%, 96% and 100%), cleared in propylene oxide and embedded in a mixture of Epon 812 and Durcupan (Sigma; polymerization for 3 days at 60°C). Semi‐thin sections were stained with toluidine blue. Ultra‐thin sections were cut on an Ultrotome Nova (LKB, Sweden), mounted into formvar carbon‐coated copper grids, counterstained with uranyl acetate and lead citrate and examined in a JEOL JEM‐1400Plus transmission electron microscope (at 120 kV JEOL, Japan). Images were captured with integrated 8Mpix CCD camera and software (JEOL, Japan).

### 2.7 Statistical analysis

Graphs were made using the GraphPad Prism 6 biostatistics software (GraphPad Software, USA). The statistical analysis between the evaluated groups was performed with one‐way ANOVA followed by a post‐hoc Tukey test (*P* < 0.05).

## 3 RESULTS

### 3.1 Proliferation and viability of BT‐549, MDA‐MB‐231 and fibroblasts after treatment with Doxorubicin and Taxol

**Figure 1:**
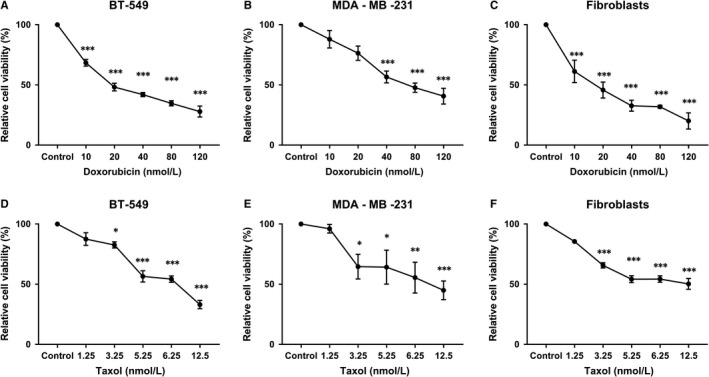
WST‐1 test after 72 h of treatment with Doxorubicin (10, 20, 40, 80 and 120 nM) in BT‐549 (A), MDA‐MB‐231 (B), and fibroblasts (C). WST‐1 test after 72 h of treatment with Taxol (1.25, 3.25, 5.25, 6.25 and 12.5 nM) in BT‐549 (D), MDA‐MB‐231 (E), and fibroblasts (F) (60×). *P < 0.05, **P < 0.01, ***P < 0.001 vs control group

Cytostatics are known inhibitors of cell viability and proliferation; therefore we had to determine a suitable concentration in which the cells would stop proliferating without losing viability. To achieve this purpose, the MDA‐MB‐231, BT‐549 and fibroblast cells were maintained with Doxorubicin or Taxol at various concentrations (10, 20, 40, 80 and 120 nM and 1.25, 3.25, 5.25, 6.25 and 12.5 nM respectively), followed by a WST‐1 test after 72 hours of treatment. The number of living cells was significantly lower after Doxorubicin (Figure 1A‐C) and Taxol (Figure 1D‐F) treatment across all cell lines.

### 3.2 Primary cilia incidence and multiciliation induced by the cytostatics Doxorubicin and Taxol

**Figure 2:**
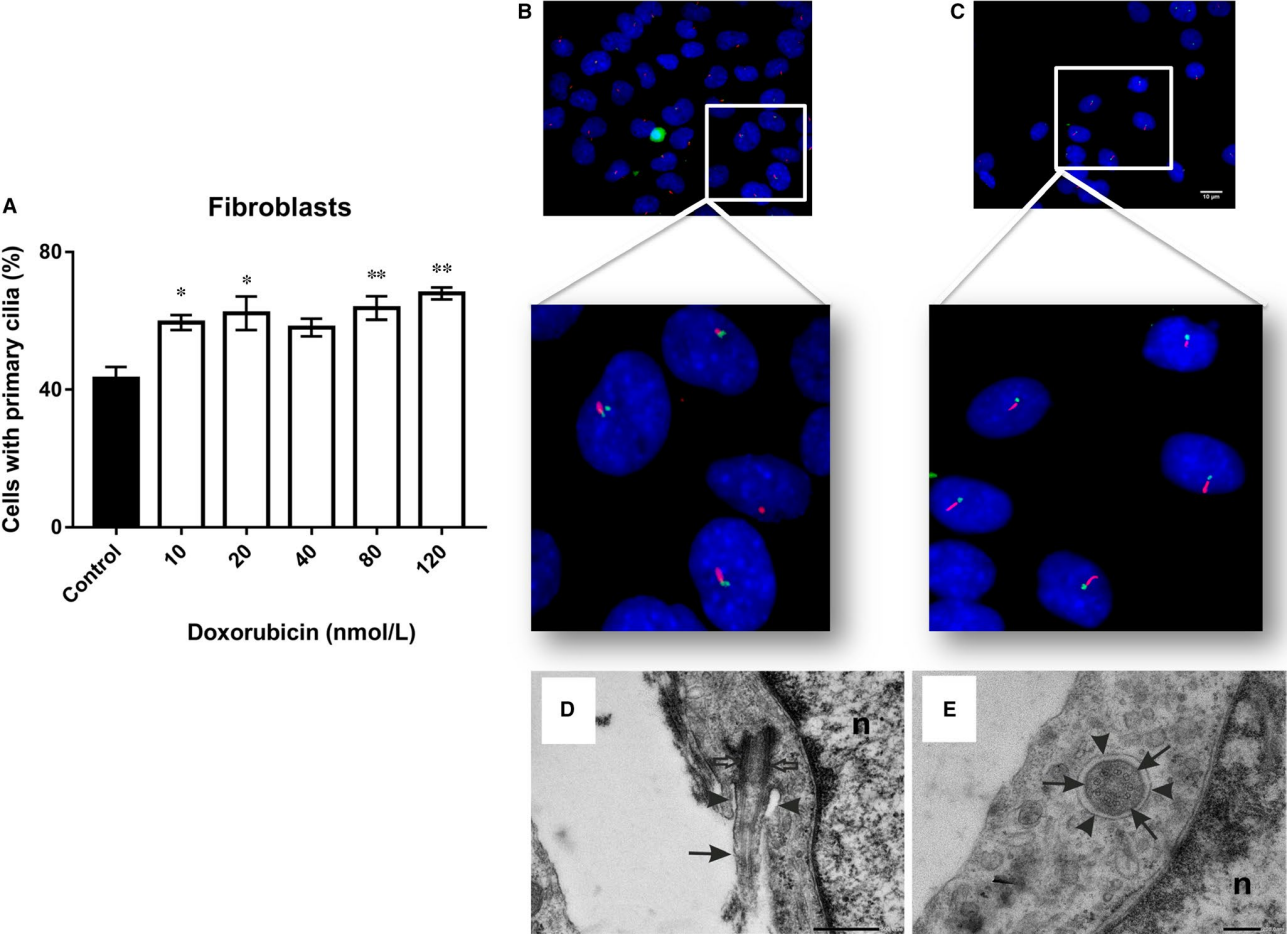
A, Shows the percentage ciliated fibroblasts after treatment with various concentrations of Doxorubicin (10, 20, 40, 80 and 120 nM). B, Control cells with primary cilia were observed after 72 h of culture. C, Primary cilia were observed 72 h after treatment with 120 nM Doxorubicin. Representative immunofluorescence images of primary cilia: acetylated tubulin (red, axoneme), gamma‐tubulin (green, basal body), nuclei (blue). D, Longitudinal section of a primary cilium (arrow) in fibroblast control cells. The cilium emerges from a basal body (open arrows). The proximal region of the cilium is situated within an invagination of the plasma membrane, a ciliary pocket (arrowheads). n, nucleus (Bar 500 nm). E, Transverse section of a primary cilium in fibroblasts after treatment with 40 nM Doxorubicin, showing the structure of its axoneme (arrows): nine microtubule doublets arranged at the periphery, an undeveloped central microtubule doublet. Outer plasma membrane of the ciliary pocket surrounding the cilium shaft (arrowheads). n, nucleus (Bar 200 nm). *P < 0.05, **P < 0.01 vs control group (60×)

**Figure 3:**
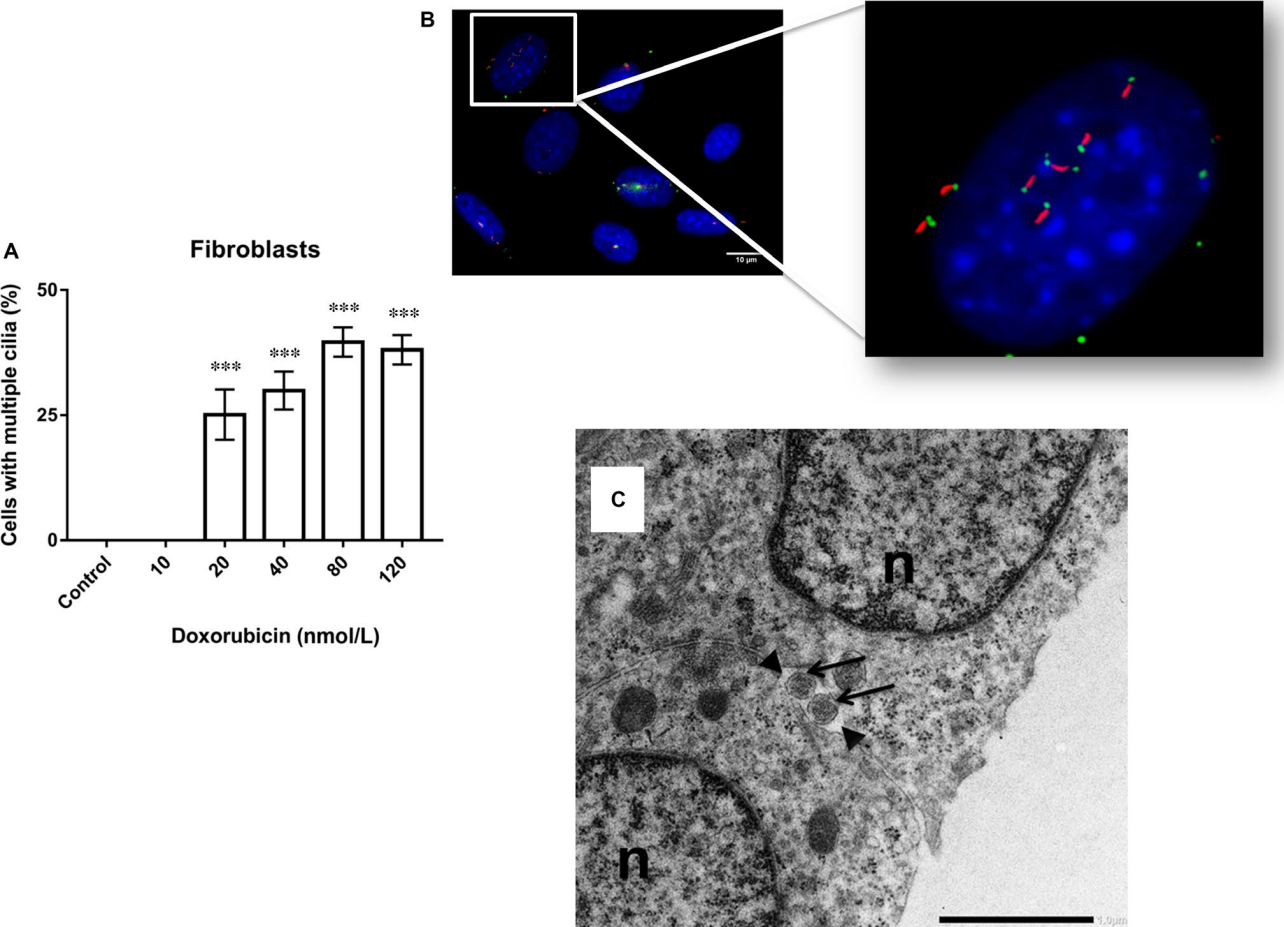
A, Shows the percentage of multiciliated fibroblasts after treatment with various concentrations of Doxorubicin (10, 20, 40, 80 and 120 nM). B, Multiple cilia were observed 72 h after treatment with 80 nM Doxorubicin. Representative immunofluorescence images of primary cilia: acetylated tubulin (red, axoneme), gamma‐tubulin (green, basal body), nuclei (blue). C, Transverse section of multiple cilia in fibroblasts after treatment with 80 nM of Doxorubicin reveals the structure of its axoneme (arrows). The outer plasma membrane of the ciliary pocket surrounds the cilium shaft (arrowheads). n, nucleus (Bar 1.5 nm). ***P < 0.001 vs control group. Magnification (60×)

Regarding Doxorubicin, the number of fibroblasts with primary cilia increased to ~70%, in comparison with control untreated cells, after 72 hours of treatment with various concentrations of this cytostatic (10, 20, 40, 80 and 120 nM), observing a higher incidence after treatment with 120 nM Doxorubicin. Overall, this effect could be observed evenly across the entire dose range used in this study (Figure 2A). After 72 hours of treatment, primary cilia were detected by immunostaining (Figure 2B – control cells; Figure 2C – 120 nM Doxorubicin) and electron microscopy (Figure 2D – control; Figure 2E – 120 nM Doxorubicin). Interestingly, ~20%‐40% of fibroblasts treated with 20‐120 nM Doxorubicin showed two or more cilia after 72 hours of treatment, observing a higher number of multiciliated cells at a dose of 80 nM Doxorubicin (~40%; Figure 3A). However, no multiciliated cells could be observed after treatment with 10 nM Doxorubicin or in untreated cells (Figure 3A). Multiple primary cilia were detected by immunostaining (Figure 3B) and electron microscopy (Figure 3C) after 72 hours of treatment.

**Figure 4:**
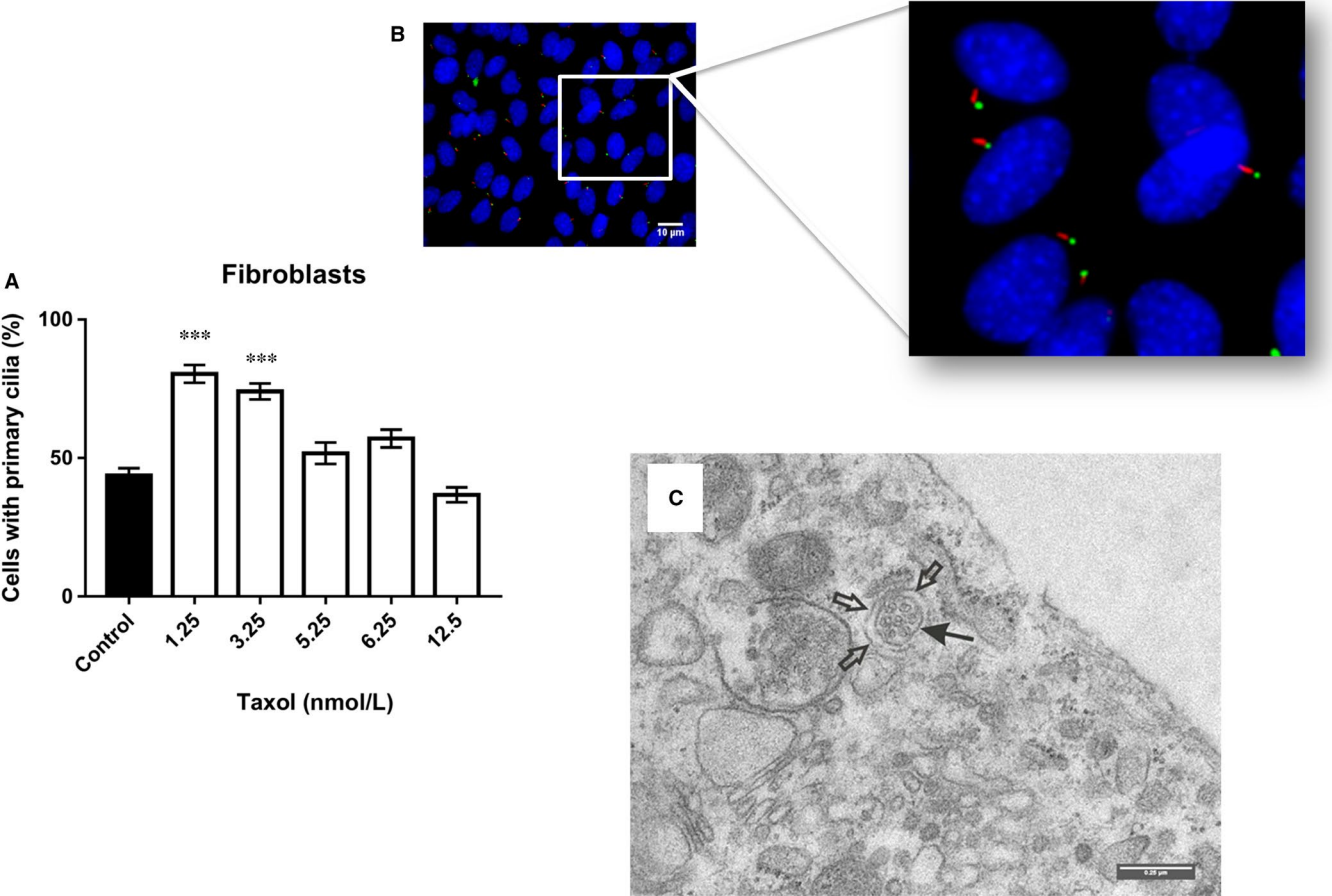
A, Shows the percentage of cells with primary cilia in fibroblasts after treatment with various concentrations of Taxol (1.25, 3.25, 5.25, 6.25 and 12.5 nM). B, Primary cilia were observed after 72 h of treatment with 1.25 nM Taxol. Representative immunofluorescence images of primary cilia: acetylated tubulin (red, axoneme), gamma‐tubulin (green, basal body), nuclei (blue). C, TEM image of fibroblasts after treatment with 1.25 nM Taxol shows the transverse section of a primary cilium (arrow), it contains six microtubule doublets arranged at the periphery and one centrally located microtubule doublet. The open arrows depict the outer plasma membrane of the ciliary pocket surrounding the cilium shaft (Bar 0.25 nm) (60×). ***P < 0.001 vs control group

Concerning Taxol, the treatment of fibroblasts with various concentrations (1.25, 3.25, 5.25, 6.25 and 12.5 nM) for 72 hours revealed a significantly higher incidence of primary cilia after treatment with 1.25 and 3.25 nM Taxol (~80%) (Figure 4A), as detected by immunostaining (Figure 4B) and electron microscopy (Figure 4C). However, this incidence rate was slightly decreased in the cells within the higher concentration range (5.25, 6.25, and 12.5 nM), although without change when compared to the untreated cells.

**Figure 5:**
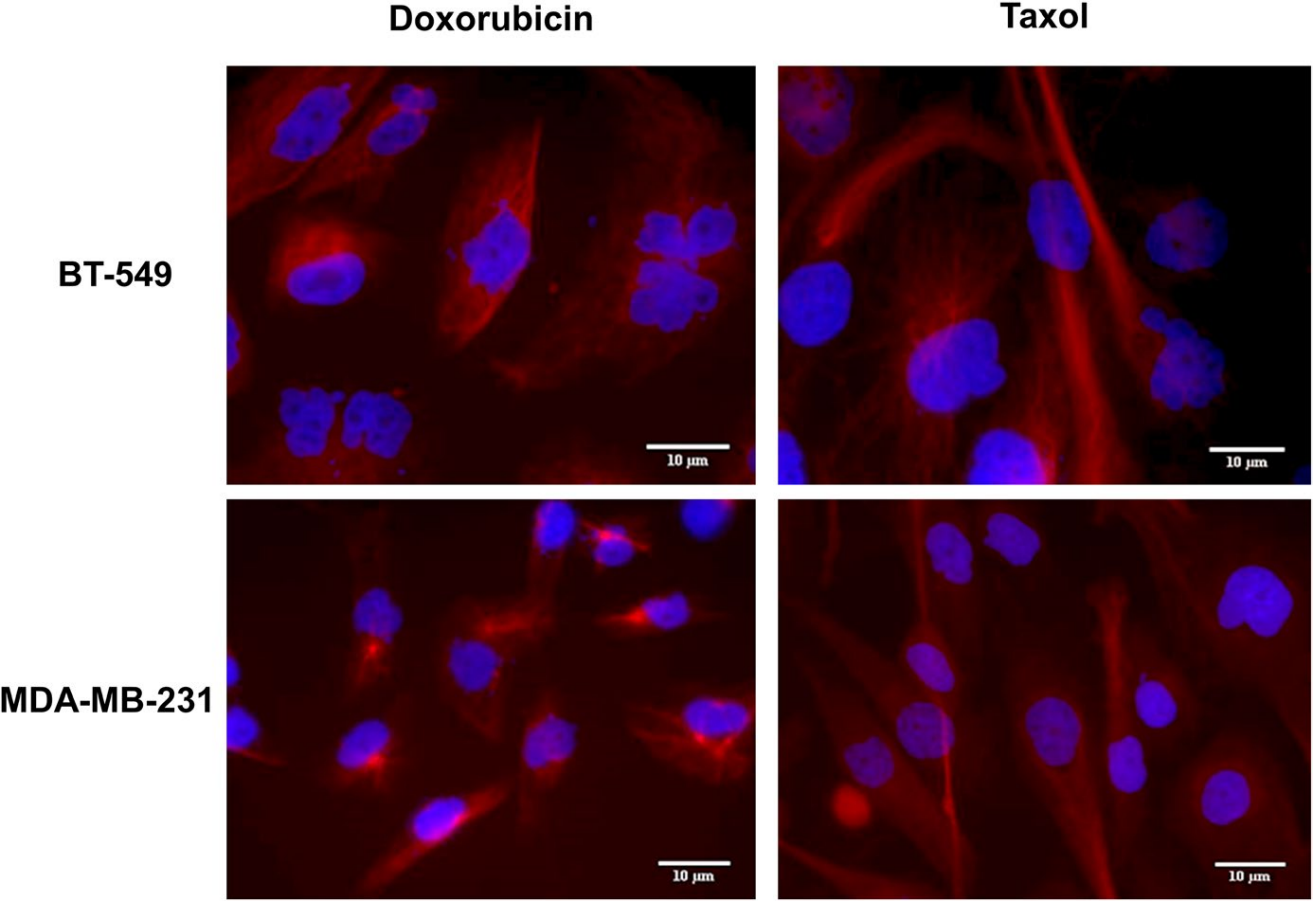
Representative immunofluorescence images of BT‐549 and MDA‐MB‐231 cells: acetylated tubulin (red), nuclei (blue). Primary cilia are absent after 72 h of treatment with Doxorubicin and Taxol in BT‐549 and MDA‐MB‐231 (60×)

Interestingly, a complete absence of primary cilia was noted in MDA‐MB‐231 cells after treatment with either Doxorubicin (10, 20, 40, 80, and 120 nM) or Taxol (1.25, 3.25, 5.25, 6.25 and 12.5 nM) for 72 hours, an effect that was repeated in BT‐549 cells (Figure 5).

## 4 DISCUSSION

In this work, we sought to determine the effect that the cytostatics Doxorubicin and Taxol could have on primary cilia incidence in triple‐negative breast cancer cells. We chose these cytostatics because they are commonly used in the treatment of breast cancer. On one hand, Doxorubicin (DOX) acts by intercalating into the DNA strands inhibiting topoisomerase II activity and thus inducing strand breaks when DNA is being replicated; in addition, it also promotes the formation of reactive oxygen species (ROS), which are highly toxic. Taxol, on the other hand, inhibits microtubule depolymerisation,^19^ resulting in shorter primary cilia and affecting their frequency in the exposed cells^15^; therefore, it was included as a negative‐control drug in our experiments.

Solid tumours possess a characteristic absence or low incidence of primary cilia which, when present, may also have a compromised structure and function. However, some tumour types that are dependent upon the Hedgehog (Hh) signalling pathway often have an increased frequency of primary cilia.^20^, ^21^ Further, several types of tumours have been associated with altered Hh, Wnt, NOTCH and Hippo signalling pathways, which are related to primary cilia; therefore, compromised signal transduction could also be caused by defects in the formation, structure or function of primary cilia.^22^

Primary cilia normally occur in approximately 70% of fibroblasts and from 7% to 19% of epithelial cells from healthy breast tissue.^11^ However, the study of 11 breast cancer cell lines revealed that primary cilia were only present in four of these and at the very low frequency of 0.3%‐4%; curiously, these cell lines had the shared characteristic of being basal B subtypes, which are analogous to triple‐negative breast cancer cells.^11^ Regardless, primary cilia have been found in some cases of TNBCs, which hints at the existence of several TNBC subtypes in which the reason for this exclusive presence of primary cilia is yet unclear. A possible explanation could be that these tumour subtypes are dependent upon the Hh signalling pathway and hence upon primary cilia, as it has been observed in most basal cell carcinomas and medulloblastomas.^22^

In this study, the chemosensitive triple‐negative breast cancer cell lines BT‐549 and MDA‐MB‐231 were used, in addition to normal skin fibroblasts as a comparative control, to test the effect that these cytostatics could have on primary cilia incidence. The cell lines were treated with 10, 20, 40, 80 and 120 nM Doxorubicin and 1.25, 3.25, 5.25, 6.25 and 12.5 nM Taxol for 72 hours to determine the dose in which the cells would become affected by the cytostatic drug without compromising viability or inducing cell death. Accordingly, the number of viable cells decreased with an increased concentration of cytostatics.

Regarding Taxol, the included cell lines showed consistently decreasing proliferation and viability as the dosage of Taxol increased; therefore, low doses of 1.25, 3.25, 6.25 and 12.5 nM Taxol were chosen based on previous experiments.^23^

In our study, fibroblasts were treated with the mentioned dose range of Doxorubicin and Taxol to establish their baseline effect on ciliogenesis. Concerning Doxorubicin, an even increment in primary cilia frequency was observed across the full dose range (10, 20, 40, 80 and 120 nM) after 72 hours of treatment, reaching a maximum value of 70% after under a 120 nM dose. Interestingly, 20% to 40% of fibroblasts showed two or more cilia after treatment with doses of 20‐120 nM, reaching the maximum value of 40% multiciliated cells after treatment with 80 nM. It must be mentioned that multiciliated cells were also in possession of multiple centrosomes, an effect that has also been observed in some tumour cells after exposure to cytostatics or ionizing radiation.^7^, ^9^ The treatment with Taxol also resulted in increased primary cilia frequency, reaching a value of 80% after 72 hours of treatment with 1.25 and 3.25 nM; however, the exposure to higher doses (5.25, 6.25, and 12.5 nM) resulted in a slightly decreased primary cilia frequency in the treated fibroblasts when compared to the lower dose range (1.25 and 3.25 nM) but not when compared against the untreated control. It must be highlighted that, unlike the treatment with Doxorubicin, the treatment with Taxol did not result in multiciliated cells.

Once the baseline effect of Doxorubicin and Taxol was determined on healthy cells, we proceeded to test their effect on primary cilia frequency in the triple‐negative breast cancer cell lines MDA‐MB‐231 and BT‐549. Unlike fibroblasts, however, the treatment of these cells with either Doxorubicin (10, 20, 40, 80 and 120 nM) or Taxol (1.25, 3.25, 5.25, 6.25 and 12.5 nM) did not increase the frequency of primary cilia, which was somewhat intriguing.

Based on previous results we were aware that primary cilia are absent or in very low percentage in the triple‐negative breast cancer cell lines BT‐549 and MDA‐MB‐231.^11^, ^24^, ^25^ This absence of primary cilia has been associated with a loss of function mutation in the tumour suppressor gene p53.^26^ However, the effect that Doxorubicin could have on primary cilia frequency in these cell lines has been heretofore unreported.

A particular issue in this regard is that multiple primary cilia are associated with an abnormal number of centrosomes in the cell and, according to other unpublished results from our group, it appears that this is a relevant issue in understanding the relation between ciliogenesis and carcinogenesis. Elaborating in this regard, several tumours and some ciliopathies^6^, ^7^, ^10^ possess a characteristic and aberrant number of centrosomes. Multiple centrosomes occur in tumour cells in which mutations of the p53, BRCA1 and BRCA2 genes are present.^27^, ^28^, ^29^ Often, the surface of cells with multiple centrosomes displays an increased number of primary cilia (2‐6) with the same structure and similar length. Curiously, the total number of Smo receptors, serotonin type 6 receptors, fibrocystin protein and Arl13b protein (ADP‐ribosylation factor‐like protein 13b) present on these multiple cilia remains the same as in single primary cilia. This suggests that since this number remains the same regardless of the number of cilia, the receptor and protein content along the total length of the primary cilia is greater, meaning that the amount of these proteins per unit of length in the primary cilia is smaller in multiciliated cases, thus resulting in a lower receptor density. Such dilution in receptor density can lead to weaker signalling stimuli,^30^ which have been observed in the alternative Wnt signalling pathways of cells with two or more primary cilia.^31^

Primary cilia play an important role in cell growth and tissue homeostasis and, in normal cells, the development of primary cilia is a dynamic process whose formation can occur in either G0/G1 or, more commonly, during the S/G2 phases; however, and almost without exception, the cilium is absorbed before entering mitosis and reappears once again in post‐cytokinetic phases of the cell cycle. This periodic cilium absorption is therefore related to the cell cycle and affects cell sensitivity to external signals associated with cilia receptors.^32^ TNBC cells, however, are notorious for their lack of primary cilia, although they can sometimes occur with extremely low frequency.^33^ Regardless, this low‐primary cilium frequency cannot be increased under serum starvation conditions in TNBC cells, which has been a proven method in healthy cells.^11^

Our study reports the null effect of Doxorubicin or Taxol on the incidence of primary cilia in triple‐negative breast cancer cell lines BT‐549 and MDA‐MB‐231. Previous reports had only addressed the use of taxanes and their effect on ciliary length in breast cancer cell lines but not of Doxorubicin, which has not been reported elsewhere. Further, we also report the presence of multiple primary cilia in breast fibroblasts induced by Doxorubicin which, to the best of our knowledge, is now reported for the first time. The null effect of these cytostatics on primary cilia incidence in the evaluated TNBC cell lines, as opposed to their effect on healthy cells, hints at some larger mechanism at play that could be involved with the inherent characteristics of malignant cells; however, these considerations must be addressed further and more in depth before an accurate conclusion can be reached.

## CONFLICT OF INTEREST

The authors declare no conflict of interest.

## AUTHOR CONTRIBUTIONS

Alžběta Filipová and Daniel Diaz Garcia wrote the article. Alžběta Filipová performed experiments and analysed the data. Daniel Diaz Garcia edited the article. Aleš Bezrouk and Dana Čížková performed experiments and analysed the data from TEM. Stanislav Filip designed the research. Josef Dvořák, Justin Sturge and Zuzana Šinkorová assisted with the design of experiments. All authors read and approved the final manuscript.
