# CORRIGENDUM

**DOI:** 10.1111/jcmm.16839

**Published:** 2021-09-05

J Cell Mol Med. 2019 Aug, 23(8):5728‐5736. https://doi.org/10.1111/jcmm.14487

**The toxic effect of cytostatics on primary cilia frequency and multiciliation**

Alžběta Filipová, Daniel Diaz Garcia, Aleš Bezrouk, Dana Čížková, Josef Dvořák, Stanislav Filip, Justin Sturge, Zuzana Šinkorová.

We, the authors, wish to amend the list of authors in our research article to include Dr. Govindan Dayanithi as follows:

Alžběta Filipová, Daniel Diaz Garcia, Aleš Bezrouk, Dana Čížková, Josef Dvořák, Stanislav Filip, Justin Sturge, Govindan Dayanithi*, Zuzana Šinkorová.

*Molecular Mechanisms in Degenerative Diseases Laboratory, MMDN, University of Montpellier, EPHE‐Sorbonne, INSERM, UMR‐S1198, Place Eugène Bataillon, F‐34095 Montpellier Cedex 5, France.

Due to loss of communication, we neglected to include his name among the list of authors and hereby amend our mistake. Dr. Dayanithi was essential in the elaboration of this manuscript and provided insightful advice during the development of this work. We are thankful for the time and effort he dedicated to this endeavour.

The online version of the article has been corrected.

